# Utilization Status and Satisfaction with Medical Services in Nonresidential Foreign Medical Tourists Visiting a Korean Medicine Hospital

**DOI:** 10.1155/2018/6586352

**Published:** 2018-05-03

**Authors:** Jaekwon Shin, Yoon Jae Lee, Joon-Shik Shin, Jinho Lee, Haneul Kim, Me-riong Kim, In-Hyuk Ha

**Affiliations:** Jaseng Spine and Joint Research Institute, Jaseng Medical Foundation, Seoul, Republic of Korea

## Abstract

Medical tourism refers to international patient travel with the intent of receiving medical care. Recently, South Korea, armed with a dual medical system of conventional and traditional Korean medicine, has been gaining international standing in this industry. This study examined the characteristics, medical service use, and satisfaction of foreign patients who visited a spine-specialty Korean medicine hospital as musculoskeletal disorders are the highest frequency category of medical conditions treated using Korean medicine. The electronic medical records of 1,733 foreign patients who had first visited an integrative Korean medicine hospital in 2012–2015 were analyzed, and a satisfaction survey was conducted by e-mail along with phone calls and interviews. Female patients in their 40s with low back or neck pain comprised the most prevalent patient group. The most frequently used visiting channels were agencies, followed by recommendation by friends or family. Patients received an average of 5.25 sessions, and, based on analysis of 134 survey results, the highest satisfaction rates were associated with acupuncture and pharmacopuncture of provided treatments, high physician expertise, and reliability among medical services and coordinating and translating services among nonmedical factors. Overall, 90.2% replied that they were satisfied and 76.9% that their perception of Korean medicine had improved following treatment. Nonresidential foreigners who received integrative medicine treatment expressed high satisfaction, but visiting and promotion channels were shown to be limited, which connotes both the potential of Korean medicine in propelling Korea forward in the global medical tourism industry and the need for more systematic promotion of Korean medicine medical tourism.

## 1. Introduction

Medical tourism refers to the practice of patients' international travel across borders with the primary intention of receiving medical care [1, 2]. Longer life expectancy, increased income, and improved quality of life as a result of global economic development have increased general interest in health and health-conscious behavior. These changes in demographic and socioeconomic climates have not only raised consumer interest in healthcare and health management but also increased awareness of disparities in medical costs and services across countries and regions as a consequence of increasingly global outlooks. Medical service providers have also been exploring new profit models that depart from existing systems. Medical tourism has recently been attracting intensified attention as a promising industry with high growth potential, as it combines high-quality medical service and tourism as a means of active healthcare and health enhancement [3].

Many countries are investing in and promoting medical tourism as a potential growth engine, and South Korea is one of the more notable countries in this regard [4]. The primary countries of residence for medical tourists of medical institutions (including both conventional and Korean medical institutions) in South Korea are the US, China, Japan, Russia, and Mongolia and visits are made based on recommendations from acquaintances, agencies, and online information for receiving internal medicine treatment, plastic surgery, dermatological treatment, or health examinations. Korean medical law was revised in May 2009 to allow visa issuance specifically for medical tourism, and medical tourism to South Korea has since increased by 1,292.4% from 7,900 patients in 2007 to 110,000 patients in 2011 [5]. Further goals to attract 1,000,000 medical tourists to South Korea by 2020 have been set [6, 7]. According to statistics on attracting foreign patients by the Korea Health Industry Development Institute in 2011, revenue from treatment via medical tourism was roughly 54,700,000,000 won in 2009, 103,200,000,000 won in 2010, and 181,000,000,000 won in 2011, showcasing a rapid annual increase [8]. However, in 2010, Thailand, India, and Singapore—countries that currently lead the medical tourism industry—attracted 1,560,000, 730,000, and 720,000 medical tourists, respectively, whereas South Korea attracted a mere 80,000 patients in comparison [9] and with a relatively low market share, which suggests the need for systematic support and improved competitiveness of its medical tourism industry.

Traditional Korean medicine and conventional allopathic medicine coexist in South Korea, each with independent and exclusive practice rights. Korean medicine doctors primarily provide traditional Korean medicine treatment using acupuncture, moxibustion, herbal medicine, and Chuna manual therapy and Korean medicine clinics and Korean medicine hospitals provide such medical services. Conventional and Korean medicine clinics hold <30 beds for inpatient care, and secondary facilities including Korean medicine hospitals hold 30≤ and <500 beds for inpatient care, and at least 4 outpatient departments including specialties. According to the Korean Health Panel Survey (KHPS) analysis of 2008-2009, musculoskeletal disorders comprised 32.3% of outpatients who received Korean medicine treatment. Musculoskeletal disorders make up a very common group of disorders and comprised up to 68.0% of national health insurance claims in Korean medicine from 2007 through 2009 [10, 11].

According to* Medical Tour of Korea 2012* published by the Korean Tourism Organization, the services that tourists wanted to use upon revisiting were medical skincare (47.9%), health check-ups (35.7%), and Korean medicine treatment (35.0%), which points to the potential of Korean medicine for increasing medical tourism rates to South Korea [9]. Indicative of this high level of patient satisfaction, medical tourism for Korean medicine has exhibited the highest growth rate out of all medical tourism sectors with an annual average growth rate of 127.2% [8]. As Korean medicine plays a significant role in attracting more medical tourists to South Korea, it could help further expand its growth to become an advanced medical tourism country.

Earlier research has discussed the general status of and satisfaction with medical tourism in South Korea, but few studies have focused on the differential characteristics of medical tourism for Korean medicine in comparison to medical tourism to South Korea in general. The few studies that were conducted on medical tourism to South Korea focused mainly on the most commonly administered treatments or were survey studies on medical tourist recipients of Korean medicine treatment. Therefore, with a wider scope, the objectives of this study were to examine the characteristics, visiting channels, treatment types, and satisfaction of nonresidential foreign medical tourists who visited a spine-specialty Korean medicine hospital for treatment of musculoskeletal disorders and to thus provide a more comprehensive overview of medical tourism for Korean medicine and examine sectors that require improvement for further promotion of this growing industry.

## 2. Materials and Methods

The participants of this study were nonresidential foreign medical tourists who visited South Korea for nonsurgical treatment at a spine-specializing integrative Korean medicine hospital. Demographic characteristics, major disorder group, visiting type, treatment costs, and visiting tendencies were analyzed by country. Treatment frequency and satisfaction with individual Korean medicine treatment types were analyzed by means of electronic medical records (EMRs) and a survey.

### 2.1. Study Setting

Designated as a spine-specialty hospital by the Korean Ministry of Health and Welfare, Jaseng Hospital of Korean Medicine is the largest Korean medicine institution in Korea and treats more than 900,000 spinal disorder cases annually [12]. Musculoskeletal disorder patients were the main focus population of this study as musculoskeletal disorders are the most common category of disorders treated using Korean medicine. Of the 10 highest frequency diagnosis codes for Korean medicine in 2016, 9 were related to “diseases of the musculoskeletal system and connective tissue (M)” or “injury, poisoning and certain other consequences of external causes (S)” in outpatients, with highest frequency for “dorsalgia (M54),” while these 2 prefix codes (M, S) ranked 1st to 6th in inpatients, with “diseases of the circulatory system (I)”, “diseases of the nervous system (G)”, and “mental and behavioral disorders (F)” codes ranking 7th to 10th. Of the 10 highest frequency diagnosis codes for Korean medicine hospitals in 2016, “dorsalgia (M54)” was most prevalent with 164,620 patients diagnosed with M54, followed by “dislocation, sprain and strain of joints and ligaments of lumbar spine and pelvis (S33),” “other intervertebral disc disorders (M51),” and “other soft tissue disorders, not elsewhere classified (NEC) (M79)” in descending order [13]. Jaseng Hospital of Korean Medicine is an integrative Korean medicine hospital where Korean medicine doctors take on a leading role in treating patients, and conventional medical doctors provide support with diagnostic imaging, handling emergency cases, and regulating acute pain with injections or conventional medication as needed. More integrative and comprehensive treatments are made available compared to Korean medicine-exclusive providers and this method of collaborative treatment is suggested to be a successful integrative treatment model in a spine-specialty Korean medicine hospital setting [14].

The hospital operates an international treatment center for assisting foreign patients, with translating coordinators equipped with medical knowledge providing accommodation and treatment reservation and aftercare services in four major language groups: English, Russian, Japanese, and Mongolian. Coordinators receive medical education on a regular basis for better patient service.

### 2.2. Patient Population

The study population was foreign patients who newly visited Jaseng Hospital of Korean Medicine located in Seoul, South Korea, between 2012 and 2015 and the study population was set as all-inclusive of nonresidential foreign patients for extensive analysis of this population group.

#### 2.2.1. Inclusion Criteria

1. All foreign patients who were recorded to have newly visited the hospital between 2012 and 2015 in the EMR database.

#### 2.2.2. Exclusion Criteria

1. Revisit and new revisit patients (patients revisiting the hospital after a discontinuance period of ≥6 months or with a new and/or different chief complaint since their last visit are administratively classified as new revisits)
2. Patients residing in Korea as their main country of residence or subscribers of Korean national health insurance
3. Patients who received only conventional medical examination for diagnosis purposes without treatment.

Patients with erroneous information or missing data in their EMRs were additionally excluded to yield a total of 1,733 patients for analysis of the characteristics and treatment history through patient chart review. To investigate satisfaction with Korean medicine treatment, these 1,733 patients were categorized into the following four survey groups: Russian (Russia, Uzbekistan, Kazakhstan, and Kyrgyzstan), Japanese (Japan), Mongolian (Mongolia), and English (the United States, Europe, the Middle East, and other countries which use English as a common language). A satisfaction survey of new foreign patients who visited between 2012 and 2015 was conducted, and later included 2016 visitors in an attempt to collect more data in light of the low response rates.

**Figure 1:**
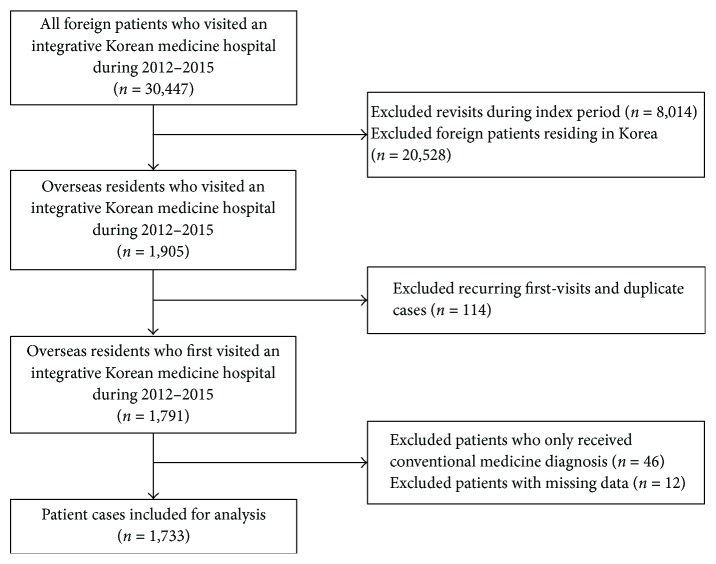
Flow diagram of the study.

Out of the 30,447 foreign patients who newly visited Jaseng Hospital between 2012 and 2015, we excluded 20,528 residential visits and 8,014 revisits. Of the remaining 1,905 visits, we excluded duplicate data and data of patients who had visited Jaseng Hospital before 2012 and revisited after ≥6 months during the index period (patients revisiting after periods of ≥6 months or with a change in chief complaint since their last visit are administratively categorized and undergo preliminary consultation as new revisit patients), which totaled 114 cases. From the remaining 1,791 visits, we excluded visits where patients exclusively sought conventional medical examination (e.g., MRI, X-ray) as they were generally not medical tourists visiting Korea for treatment purposes but general tourists seeking diagnostic examinations for mild complaints such as injuries sustained during their visit to Korea. In addition, fifty-eight visits with erroneous information (e.g., missing data) were also excluded, and the remaining 1,733 visits of nonresidential foreigners newly visiting the hospital were included for analysis (Figure 1).

### 2.3. Treatment

The main treatment modalities of Korean medicine may be largely divided into the following categories: acupuncture and electroacupuncture, bee venom pharmacopuncture and pharmacopuncture, herbal medicine, Chuna manual therapy, cupping, and moxibustion. The conventional medicine sector was categorized into conventional medicine diagnosis and treatment (radiological examinations, physical therapy, manipulation, and conventional medication), and patient charts were analyzed for treatment history of treatment interventions administered to patients with the aim of establishing high frequency Korean medicine treatments in this population. The number of hospital visits was categorized into once, 2–10, 11–20, 21–30, and >30 times.

### 2.4. Disease Codes (Administrative Codes for Diagnostic Categorization)

With regard to patient visits, the 6th revision of the Korean Standard Classification of Diseases (KCD-6), which is the Korean version of the World Health Organization's (WHO's) 10th revision of the International Statistical Classification of Diseases and Related Health Problems (ICD-10), was used to determine disease classification [15]. Up to two disease classifications were selected per patient. A total of 1,733 nonresidential foreign patients were categorized by disease classification, and the anatomical region (e.g., neck, low back, shoulder, and knee) corresponding to each disease was identified for frequency.

### 2.5. Survey

A survey was conducted to assess patient satisfaction. Earlier research has supported the importance of human resources, such as doctors and nurses [16–18], and physical infrastructure, such as facilities and environment [19, 20]. The survey used in this study consists of six questions designed to evaluate general satisfaction with Korean medicine treatment, the hospital and services provided by the hospital, preferred treatment type, and any change in attitudes towards Korean medicine after visiting. The objective was to identify the most significant factor that affected foreign patients' satisfaction when visiting an integrative Korean medicine hospital (see Supplementary Materials (available here) for the translated English version of the full contents of the survey).

The survey was e-mailed to nonresidential medical tourist patients who visited Jaseng Hospital between 2012 and 2015 and provided e-mail addresses as contact information. From August 2016 to September 2016, English, Russian, Japanese, and Mongolian translations of the survey were e-mailed to patients, and patients who did not respond to the first e-mail were sent a follow-up e-mail. Delivering the survey using only two rounds of e-mails resulted in a very low response rate, and no e-mail responses were obtained from the Mongolian patient group. In an attempt to increase the response rate, the survey was additionally conducted at the hospital immediately after patients received treatment, along with a telephone survey of patients who had since returned to their countries after treatment had concluded. For Mongolian patients, medical staff members conducted face-to-face interviews during a business trip to Mongolia.

### 2.6. Statistics

All statistical analyses were conducted with STATA 14.0 (StataCorp, College Station, Texas, USA). Continuous variables are presented as mean ± SD and categorical variables as *n* (%).

### 2.7. Ethics, Consent, and Permissions

The study protocol was approved by the Institutional Review Board of Jaseng Hospital of Korean Medicine in Seoul, Korea (IRB approval number: KNJSIRB2017-10-006), and all foreign patients gave written informed consent to the use of medical records for academic purposes upon their first visit to the hospital.

## 3. Results

Analysis of the 1,733 patient EMRs showed that 59.1% of medical tourism patients to this integrative Korean medicine hospital were women. The most prevalent age group was patients in their forties (26.7%), followed by those in their fifties (23.8%) and thirties (20.8%), respectively. The country from which the greatest number of patients visited was Japan (26.9%), followed by Russia (26.7%) and Kazakhstan (20.4%), in order of decreasing frequency. The medical tourism patients mainly visited for treatment of musculoskeletal diseases, and the most common single disease group was cervicalgia (M542).

**Figure 2:**
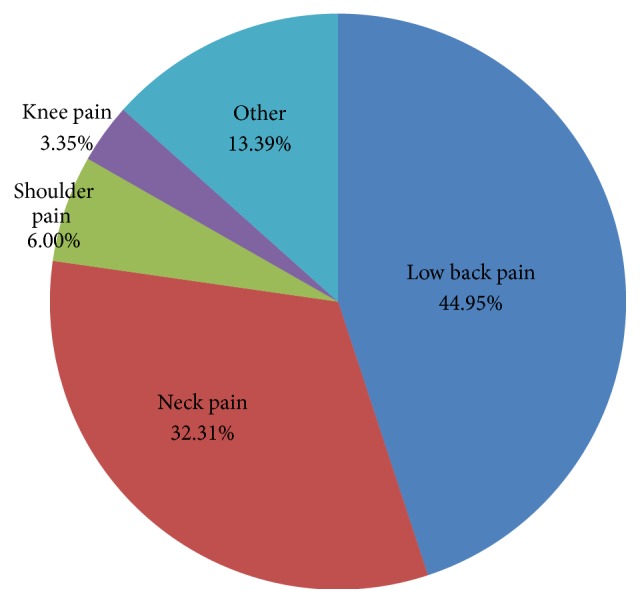
Main complaints of foreign patients.

The most frequently used visiting channel was agencies (32.1%), followed closely by recommendations (29.3%). Agencies are organizations that specialize in connecting patients with local hospitals and are issued registration certificates by institutions that promote foreign patient visits. They charge a set fee and cater to such patient needs as suggesting hospital options and assisting with visiting procedures such as visa issuance and finding interpreters. On the other hand, travel offices are companies that do not specialize in medical tourism but in general tourism, and travel offices as visiting channels refers to when tourists specifically request medical care for their condition to these offices in their country of residence or during their stay in Korea. Patients who visited based on recommendations were referred to this hospital by acquaintances or other hospitals in Korea. Online searching refers to patients' visiting the hospital after coming across hospital information in the course of actively searching for information on their medical condition. Cases in which patients were led to visit this hospital through local magazine articles or TV programs introducing Korean medical tourism were categorized as visits through advertisements. Other visiting channels were when patients themselves had prior knowledge of this hospital or events such as veteran medical care and broadcast filming (Table 1). Nearly half of the patients had back-related complaints (45.0%), while about one-third of the patients had neck conditions (32.3%) (Figure 2).

**Figure 3:**
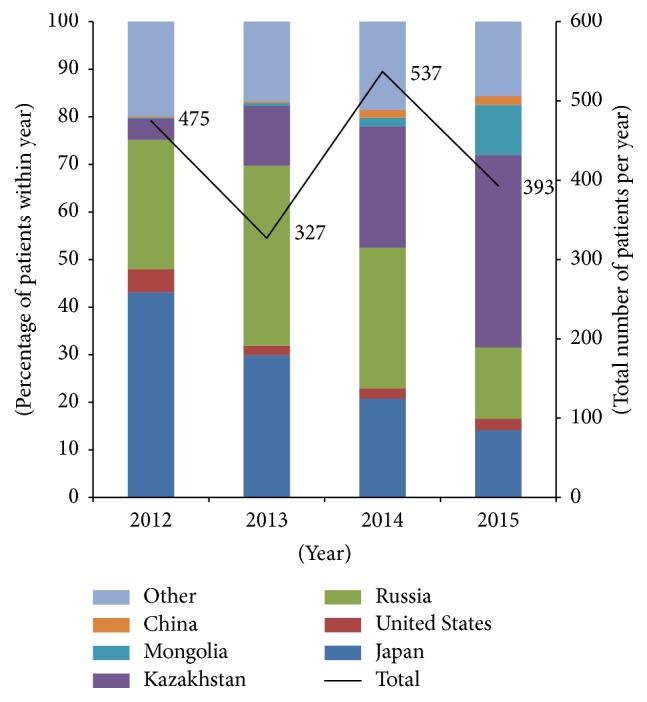
Total number and percentage of foreign patients by nationality.

Analysis of visiting tendencies by country revealed that Japan, which initially had the highest proportion of all new foreign patients in 2012 (43.2%), exhibited a decreasing annual visiting tendency and numbers dropped to 13.7% in 2015. In contrast, patient visits from Kazakhstan, Mongolia, and China increased steadily. Visits from Kazakhstan increased by 10 times and Mongolia by 50 times over the index period, showing significant increases in medical tourism to this hospital. Patient visits from the US and Russia did not show fixed trends but rather fluctuated throughout (Figure 3).

Analysis of treatment tendencies revealed that patients visited 5.25 ± 7.15 times on average and that the most common visiting frequency range was 2–10 times. Of all the traditional Korean and conventional medical examinations and treatments, the most commonly used treatment modality was pharmacopuncture (*n* = 1,289, 74.38%), which was notable in that it was used more than acupuncture (*n* = 1,267, 73.11%), albeit marginally. In conventional medicine, X-ray examination was used the most often (*n* = 710, 40.97%), followed by MRI (*n* = 374, 21.58%). Pharmacopuncture is a relatively new type of acupuncture that is used for greater effect and to obtain swifter results by combining the benefits of acupuncture and herbal medicine, the two most frequently used Korean medicine treatment methods, by mechanically and chemically stimulating acupuncture points with extracted herbal medicine. This method prolongs the effect of acupuncture and herbal medicine by optimizing acupoint access and thereby maximizing treatment efficacy [21, 22]. It can also be surmised that pharmacopuncture was used at a higher frequency to the aim of higher treatment success rates as treatment timeframes tend to be more limited in medical tourism.

The average medical expense paid for total visits by foreign patients at Jaseng Hospital was 2,262,123 ± 3,263,322 won, and the proportion of inpatient treatment was 2.0% of the total treatment types (Table 2).

We analyzed 134 survey results of nonresidential, foreign patients newly treated at this hospital between 2012 and 2016 using e-mail, telephone, and field interviews. Based on the survey, patient satisfaction with the overall medical service of Jaseng Hospital was very high, as 90.2% reported their experience to be satisfactory or higher. Korean medicine treatments were administered concurrently or selectively according to patient condition, and of these treatments, acupuncture and pharmacopuncture treatments indicated the highest satisfaction levels, and the most satisfactory hospital service factor was the specialty and reliability of doctors (73.1%), followed by coordinator and translation services (68.7%). Of all the patients who responded to the survey, 76.9% reported a positive change in their perception of Korean medicine following treatment (Table 3).

## 4. Discussion

Medical tourism requires patients to cross national borders for treatment and is fundamentally different from the medical service use patterns seen in domestic residents. Therefore, understanding the characteristics of medical tourists is essential in further developing and complementing this industry. This study analyzed 1,733 nonresidential foreign patients who newly visited an integrative Korean medicine hospital that specializes in spine diseases. The main patient group was middle-aged patients in their forties to sixties whose chief complaint was pain in the low back and neck regions who received treatment over an average of five visits during their stay in Korea. Most patients expressed satisfaction with Korean medicine, and the most satisfactory treatments were acupuncture and pharmacopuncture, while the most satisfactory medical services were the expertise of the physician and systematic explanation of the disease and posttreatment management strategies.

A total of 22,433 foreign patients newly visited this Korean medicine hospital during 2012–2015 and of these visitors 1,733 were nonresidents (7.73%). A satisfaction survey was conducted in 134 nonresidential foreign patients out of these 1,733 nonresidential visitors (7.73%). Though this data is not representative of all foreign patients visiting this medical institution, it is a current status report and analysis of foreign patients visiting from overseas for the purpose of medical tourism. Moreover, there are no studies to date investigating the current status and satisfaction of foreign patients visiting a Korean medicine hospital, and this study's main significance is that it is an analysis of several years' worth of administrative data and satisfaction in an elusive patient population.

Most medical tourism patients to the hospital were in their forties (26.7%), fifties (23.8%), and thirties (20.8%). Comparing these percentages to those of the general foreign patients who visited hospitals in Korea in 2012 who were primarily in their thirties (20.8%), twenties (21.9%), and forties (19.4%) [23], it is likely that the patients who visited Jaseng Hospital were older as the frequency of spinal diseases increases after middle age and spine and joint disorders are often accompanied by associated degenerative changes.

In 2012, the greatest number of medical tourists to South Korea (encompassing both conventional and Korean medical institutions) was from China (24.0%), followed by the US (23.0%), Japan (14.1%), Russia (14.1%), and Mongolia (6.4%) [23], while, in this study, the greatest number of patients was from Japan, Russia, Kazakhstan, Mongolia, and the US in decreasing order, which deviates from the general statistical trend of medical tourism to South Korea. This is largely due to the difference in medical specialty departments at Jaseng Hospital compared to other conventional and Korean hospitals in Korea, as those most frequently visited overall by medical tourists to hospitals in Korea were internal medicine (22.3%), medical examination (11.6%), dermatology (7.9%), plastic surgery (7.6%), obstetrics and gynecology (5.3%), orthopedics (4.7%), Korean medicine (4.6%), and ophthalmology (3.8%) [23]. Of the foreign patient nationalities, Americans usually visited South Korea for medical examination, dermatologic treatment, and plastic surgery, while Chinese patients most commonly visited for antiaging and cosmetic purposes [23]. Meanwhile, the departments that foreign medical tourists visited most frequently at Korean medicine institutions (excluding conventional medicine institutions) were internal Korean medicine, Sasang constitutional medicine, and Korean medicine obstetrics and gynecology in descending order in 2013, and internal Korean medicine, Korean medicine dermatology, and Korean Medicine Rehabilitation in 2014 and 2015 [24]. It can be suggested that the proportion of nationalities of visitors to Jaseng Hospital of Korean Medicine is different as it is a spine-specialty Korean medicine hospital that specializes in musculoskeletal disorders. Though Chinese patients constituted the greatest number of total medical tourists to South Korea, only 1.2% visited Korean medicine hospitals/clinics [25], similar to the 1.0% of Chinese medical tourists who visited the hospital in this study. This may be due to the fact that Chinese patients are more likely to seek traditional Chinese medicine services in China. Other than China, Russian, Japanese, and Kazakhstani patients are geographically proximal to and share a strong Asian cultural background with South Korea compared to Western European countries and are therefore relatively more familiar with Korean medicine, which likely explains the higher prevalence in this study.

We identified the countries from which the greatest number of patients visited Jaseng Hospital to analyze the main countries and their statistics and compared them to those most involved in general medical tourism to South Korea. Japan, which had the highest percentage of new foreign patient visits to this hospital in 2012 (43.2%), exhibited a gradual decrease from 12.4% in 2012 to 6.4% in 2015 in general Korean medical tourism statistics [25], a trend that may be attributed to the deflation of the Japanese yen to Korean won currency exchange rate. Various efforts are being directed towards overcoming this environmental drawback such as active participation at local medical exhibitions and increased promotion through use of magazines and agencies. Russian patient visits increased from 27.2% in 2012 to 35.2% in 2013 due to active strategies such as advertisement campaigns and medical exchanges with Russia. In 2014, the relative proportion of Russian patients decreased to 30.2%, but the actual number increased from 115 in 2013 to 162 in 2014. However, in 2015, the number of visits did not even reach half of that of 2014 due to the fall of the Russian rouble exchange rate towards the end of 2014. This tendency is also reflected in the total Korean medical tourism patients from Russia from 2012 through 2015 (10.3% (2012), 11.4% (2013), 11.9% (2014), and 7.0% (2015)) [25]. The total number of medical tourists from Mongolia increased in 2012, but the visits gradually decreased starting from 2013 (5.3% (2012) to 4.2% (2015)) [25]. The percentage of Mongolian patients who visited Jaseng Hospital significantly increased by about 50 times, from 0.2% in 2012 to 10.2% in 2015. The total number of Kazakhstani patients increased in light of the visa waiver program (1.0% (2012) to 4.2% (2015)) [25], and Kazakhstani patient visits to Jaseng Hospital also exhibited a sharp increase from 4.4% in 2012 to 39.2% in 2015, equaling a nearly 9-fold increase. This is comparable to the situation in Russia where active publicity and promotion heightened local awareness, and patients were exposed to various opportunities to experience and familiarize themselves with Korean medicine treatment through such promotional activities as local medical volunteer work. China and the US have the highest and second highest number of foreign medical tourists who visit South Korea, respectively, and the number of visiting patients is on the rise. From these results, we can confirm the close relationship between political support—such as a favorable currency exchange rate, the country's economic situation or strength, and visa waiver programs—and overseas patient visits.

Analyzing the methods by which medical tourists obtain information about hospitals and the channels through which they visit hospitals can form the basis for understanding and determining the most effective marketing angle to increase medical tourism. In the context of overall medical tourism to South Korea, foreign patients were shown to obtain information about Korean medical tourism through recommendations from acquaintances (39.6%), online searches (22.9%), agencies (19.3%), and advertisements in magazines and broadcasting (15.5%) [24]. However, in this study, more patients visited through the help of agencies and fewer patients visited based on the recommendation of acquaintances and magazine and TV advertisements compared to other Korean hospitals. This result indicates that traditional Korean medicine and integrative medicine treatment is relatively less accessible and limited in reaching potential medical tourists and foreign patients, an urgent problem that should be prioritized in strengthening its standing. We additionally analyzed the influx of foreign patients from 2012 to 2015 but did not find significant monthly or seasonal differences (data not shown). This may suggest the possibility of attracting medical tourists by capitalizing on Korea's unique natural, seasonal, and cultural resources and correlating them with medical tourism to Korea.

Cervicalgia (M542) was the most prominent single disease group (Table 1), but, overall, low back conditions (45.0%) were more common than those related to the neck (32.3%) (Figure 2). Figure 2 shows that 13.4% of patients were treated for miscellaneous diseases, including diseases of the hip joint, internal medicine, or treatment for cosmetic purposes (e.g., antiaging).

The reason why the most common number of visits is 2–10 times, as shown in Table 2, most likely has to do with the nature of medical tourism, the purpose of which is to focus on short-term treatment. While various treatment modalities may be selected for individualized treatment in observance of patient condition and clinical symptoms, they are generally performed concurrently and in a more condensed manner in medical tourism to maximize treatment effects and yield optimal results within a shorter timeframe. Patients usually stayed in Korea for seven days or less (48.3%) or eight to fourteen days (30.1%) [24].

Across Korean hospitals, the overall average medical expenses paid by a foreign patient for total visits was 1,040,000 won in 2012 [26] compared with 2,262,123 ± 3,263,322 won at Jaseng Hospital, which is quite high even after taking the inflation rate into consideration. At Jaseng Hospital, 2.0% of foreign patients received inpatient treatment for an average of 15.63 ± 14.45 days in 2012, of which the percentage is less than the 9.3% of foreign inpatients across all Korean hospitals [26]. This may be because most treatments were provided to outpatients, given that patients mainly complain of chronic rather than acute diseases and are relatively mobile.

Finally, we assessed the satisfaction of patients following treatment. Patient satisfaction is of heightened importance in medical tourism as a significant investment of patients' time and money. Moreover, patient satisfaction levels are barometers of patient experience of medical and nonmedical services as they are influenced by service quality and indicative of revisits and future patient behavior [27]. The main purpose of this study was to investigate the characteristics and satisfaction of foreign patients who visited a Korean medicine hospital, and the survey focused on the influence of Korean medicine treatment factors on patient satisfaction.

Analysis of 134 surveys (Table 3) revealed that over 90% of patients replied that they were “satisfied” or higher with regard to their overall satisfaction. However, the possibility that satisfied patients were more likely to respond to the survey cannot be disregarded. Of hospital services, the highest levels of satisfaction were associated with the expertise and reliability of doctors and with coordinator and translation services. Satisfaction with the reliability of doctors and staff have been reported to play an important role in the revisiting rate for medical tourism in a study by Han and Kim [28]. The high satisfaction levels with the hospital's professional human resources also led to a positive change in perception of Korean medicine following treatment (76.9%). It is worth noting that acupuncture and pharmacopuncture were the most satisfactory treatments despite being relatively painful and unfamiliar to nonresidential foreigners.

Limitations of this study include the following points. The survey was sent to patients via e-mail, but some countries have limited Internet access, and errors in patient-provided e-mail addresses contributed to the nonresponse rate. Investigating the satisfaction levels and intention of medical tourists is not simple, as noted by many other studies, due to the lack of data and empirical analysis references and the difficulty of obtaining responses from medical tourists [29, 30]. As Jaseng Hospital of Korean Medicine provides patient care according to a standardized treatment protocol, treatment methods and patient care during this period were considered to be comparable in their homogeneity, and the researchers additionally collected survey results from foreign patients newly visiting in 2016 to increase the sample size when the response rate for new visitors for 2012–2015 did not meet expectations. This method helped the study partially overcome its conditional difficulties but made the research method inconsistent. Additionally, although the authors reviewed numerous surveys in creating the questionnaire, it still suffers methodological limitations as its validity and credibility were not verified and the survey is very brief in an effort to increase response rate. Future studies should consider including survey items examining satisfaction of specific medical and nonmedical supporting services provided by the hospital and additional qualitative interview surveys to gauge deeper understanding of the overall medical tourism experience and issues for improvement. This study also lacks assessment of clinical and functional measures as a study mainly based on hospital administrative records (i.e., EMRs), and the study survey was conducted by collecting the e-mail addresses of 1,733 patients and sending out survey links by e-mail to maintain anonymity and protect personal information. This precludes additional analysis of survey results by patient characteristics to further determine clinical implications and comprises an additional limitation of the study. Furthermore, though musculoskeletal diseases are the most common condition treated with Korean medicine, this study does not paint the full picture for medical tourism to Korea medicine institutions as the external validity cannot be ensured, restricting its scope and generalizability and thus limiting the data's representativeness.

There is also the possibility that patient complaints may have been recorded differently at the physician's discretion or for administrative reasons, and certain patient charts were missing information such as disease classifications and therefore had to be excluded from the sample. Lastly, despite efforts towards objective data analysis and deduction of conclusions, this study is flawed in that it holds the limitation of being conducted at the same institution where the authors are employed and therefore it is not independent in analyses and reporting of results, albeit similar to most other usage and satisfaction reports.

Certain traditional medicine disciplines have been receiving increasing interest as an alternative to conventional medicine with respect to its limitations, and the efficacy and satisfaction of Korean medicine for treating various conditions including musculoskeletal diseases have been studied. However, few studies have been conducted on Korean medicine in regard to medical tourism. In the competitive and multidimensional medical tourism market, the aim of this study was to help improve the overall quality of medical tourism services by providing a window into concurrent real-world Korean medicine medical tourism and to suggest a new integrative treatment model to Korean medicine hospitals comparable to the study setting by analyzing factors associated with satisfaction in foreign patients who visited Jaseng Hospital for integrative medicine treatment with focus on nonsurgical Korean medicine treatment. Future studies may consider research on clinical outcome measures such as the status, satisfaction, visiting channels, and symptom improvement in revisiting medical tourists to further identify medical tourist patient characteristics and their responsiveness to treatment.

## 5. Conclusion

Satisfaction with Korean medicine treatment was high among nonresidential foreigners who experienced integrative medicine treatment at a Korean medicine hospital that specializes in musculoskeletal disorders, which emphasizes the potential of Korean medicine and the integrative treatment model in medical tourism. This study is hoped to be of use to medical tourism professionals and related policy-makers in further understanding, promoting, and improving medical tourism to South Korea as visiting and promotion channels were limited.

## Figures and Tables

**Table 1:** Characteristics of foreign medical tourism patients visiting an integrative Korean medicine hospital.

Factor	Mean ± SD	*n*	(%)
Sex			
Male		709	40.91
Female		1024	59.09
Age	45.30 ± 14.08		
<20		59	3.4
20≤, <30		168	9.69
30≤, <40		361	20.83
40≤, <50		462	26.66
50≤, <60		413	23.83
60≤, <70		199	11.48
70≤		71	4.1
Country			
Japan		466	26.89
Russia		463	26.72
Kazakhstan		353	20.37
Mongolia		53	3.06
US		50	2.89
Germany		21	1.21
Saudi Arabia		21	1.21
Uzbekistan		21	1.21
Unknown		76	4.39
Other		209	12.11
Main diagnosis^a^			
M542		369	21.29
M545		346	19.97
M511		271	15.64
M255		123	7.1
M179		93	5.37
M501		83	4.79
Z00		44	2.54
M544		40	2.31
Other		364	21.1
Visiting channel			
Recommendation		508	29.32
Online search		355	20.49
Agency		551	31.79
Travel office		95	5.48
Advertisement		54	3.11
Other		170	9.81

^a^M542, cervicalgia; M545, low back pain; M511, lumbar and other intervertebral disc disorders with radiculopathy; M255, pain in joint; M179, gonarthrosis, unspecified; M501, cervical disc disorder with radiculopathy; Z00, general examination and investigation of persons without complaint or reported diagnosis (mainly for cosmetic purposes (i.e., antiaging)); M544, lumbago with sciatica.

**Table 2:** Treatment details for foreign medical tourism patients visiting an integrative Korean medicine hospital.

	Mean ± SD	*n*	(%)
*Outpatient visits*			
Average number of visits	5.25 ± 7.15		
1		691	39.87
2≤, ≤10		799	46.11
11≤, ≤20		183	10.56
21≤, ≤30		38	2.19
>30		22	1.27
*Treatment*			
Lab test	1.12 ± 0.42	219	12.64
X-ray	1.69 ± 0.97	710	40.97
CT	1.2 ± 0.42	10	0.58
MRI	1.36 ± 0.89	374	21.58
Acupuncture	6.39 ± 7.87	1,267	73.11
Electroacupuncture	6.33 ± 5.89	642	37.05
Pharmacopuncture	6.4 ± 8.87	1,289	74.38
Bee venom pharmacopuncture	7.67 ± 5.41	21	1.21
Chuna manual therapy	6.41 ± 7.70	1,100	63.47
Herbal medicine (days of prescription)^a^	35.42 ± 56.00	199	11.48
Cupping therapy	10.58 ± 12.43	447	25.79
Manipulation	5.7 ± 10.41	192	11.08
Simple physiotherapy	6.84 ± 6.06	358	20.66
Conventional medication	1.21 ± 0.66	140	8.08
*Expenditure*			
Total expenses (Korean won)	2,262,123 ± 3,263,322		
Expense per visit (Korean won)	457,596.2 ± 617,144.1		
*Hospitalization*			
Inpatients		35	2.02
Outpatients		1698	97.98
Days of hospitalization	15.63 ± 14.45		

CT, computed tomography; MRI, magnetic resonance imaging. ^a^The main herbal medicine prescribed was Chungpa-jun, of which the main herbal ingredients are *Ostericum koreanum*, *Eucommia ulmoides*, *Acanthopanaxsessiliflorus*, *Achyranthes bidentata*, *Psoralea corylifolia*,* Peucedanum japonicum*,* Cibotium barometz*, *Lycium chinense*,* Boschniakia rossica*,* and Cuscuta chinensis*.

**Table 3:** Satisfaction survey conducted in foreign medical tourism patients visiting an integrative Korean medicine hospital (*n* = 134).

	*n*	%
*Satisfaction level with Korean medicine treatment*		
Very satisfied	76	57.14
Satisfied	44	33.08
Neutral	10	7.52
Unsatisfied	2	1.5
Very unsatisfied	1	0.95
*Most satisfactory Korean medicine treatment (multiple responses allowed)*		
Acupuncture	63	47.01
Pharmacopuncture	62	46.27
Chuna manual therapy	53	39.55
Herbal medicine	55	41.04
No response	3	2.24
*Most satisfactory medical service (multiple responses allowed)*		
Hospital facilities and equipment	33	24.63
Expertise and reliability of the attending physician	98	73.13
Friendliness/courtesy of the medical staff	52	38.81
Cost-effectiveness of the treatment provided relative to one's own country	22	16.42
Assistance and interpretation provided by the assisting staff	52	38.81
Time-sensitivity of the medical services	45	33.58
*Most satisfactory nonmedical service (multiple responses allowed)*		
Information given regarding patient condition, treatment, and self-care methods	77	57.46
Explanation of the total and individual item costs	14	10.45
Services provided in consideration of cultural and religious diversity	11	8.21
Coordinator and interpretation services	92	68.66
Convenience and appropriateness of complaint filing and handling	9	6.72
Others	8	5.97
*Change in perception of Korean medicine following use*		
Better	103	76.87
Worse	2	1.49
Same	26	19.4
No response	3	2.24

## Supplementary Materials

Supplementary Materials Supplement (PDF file): translated English language version of full survey contents.
