# Supplementary material for: Utilization Status and Satisfaction with Medical Services in Nonresidential Foreign Medical Tourists Visiting a Korean Medicine Hospital
Source: Evid Based Complement Alternat Med. 2018 May 3;2018:6586352. doi: 10.1155/2018/6586352 (PMC5960560; doi:10.1155/2018/6586352)
Supplement: Supplementary Materials — Supplement (PDF file): translated English language version of full survey contents. [file 6586352.f1.pdf]

Hello.
The employees of Jaseng Hospital of Korean Medicine are dedicated to providing our patients with the best care possible.
This questionnaire was put together to evaluate our overall performance. The questionnaire is expected to take only a few minutes of your time, and the results will help us further improve our services and make amendments for any shortcomings.
Your contribution is greatly appreciated. Thank you in advance.

1. Are you satisfied with the overall Korean medicine treatment(s) provided at Jaseng?

Response: (   )

1) Very satisfied 2) Satisfied 3) Neutral 4) Unsatisfied 5) Very unsatisfied

2. In your experience, which treatment method was most satisfactory?

Response: (   )

1) Acupuncture 2) Pharmacopuncture 3) Chuna manual therapy 4) Herbal medicine

3. In your experience, which of the following services provided by the hospital was most satisfactory?

Response: (   )

1) Hospital facilities and equipment
2) Expertise and reliability of the attending physician
3) Friendliness/courtesy of the medical staff (i.e., physicians, nurses)
4) Cost-effectiveness of the treatment provided relative to your home country
5) Assistance and interpretation provided by the assisting staff (i.e., coordinators)
6) Time-sensitivity of the medical services (i.e., diagnosis, treatment, consultation) provided

4. Aside from the treatment, which of the following services did you find to be satisfactory?

Response: (   )

1) Information on your condition, treatment, and self-care methods
2) Explanation of the total and individual item costs
3) Services provided in consideration of cultural and religious diversity

4) Coordinator and interpretation services

5) Convenience and appropriateness of complaint filing and handling

6) Other(s)

5. Compared to before receiving treatment in Korea or learning about Jaseng treatment, how has your perception of Korean Medicine changed after receiving treatment at Jaseng?

Response: (   )

1) I am more favorably disposed towards Korean Medicine

2) I am less favorably disposed towards Korean Medicine

3) My perception of Korean Medicine has not changed

6. Please feel free to leave any additional comments you have about Jaseng Hospital of Korean Medicine.

Response: (   )
